# Using the English GSAT for placement into EFL classes: accuracy and validity concerns

**DOI:** 10.1186/s40468-022-00181-6

**Published:** 2022-08-30

**Authors:** Yen-Fen Liao

**Affiliations:** National Taiwan University Department of Foreign Languages and Literatures, National Taiwan University 1 Section 4, Roosevelt Road, Taipei, 106 Taiwan

**Keywords:** Validation, EFL placement test, College entrance exam, The English General Scholastic Ability Test, The General English Proficiency Test

## Abstract

The English General Scholastic Ability Test (GSAT), a nationwide high-stakes college entrance exam in Taiwan, has commonly served as a placement test for streaming college freshmen into EFL classes. There has, however, been scant research reported on the feasibility of using an admission test for EFL placement. This study aims to investigate the accuracy and validity of placement decisions being made using the English GSAT scores, with a focus on its relations with the General English Proficiency Test (GEPT) and students’ subsequent performance in class. Quantitative data, including the English GSAT scores, the GEPT High-Intermediate Listening and Reading scores, and the English final course grades, were collected and statistically analyzed from four EFL classes with a total of 100 students at a university in Taiwan. The English GSAT was found to function well to place students into distinctively lower or higher level of EFL classes but did not discriminate well for the borderline cohort. A three-level placement using the English GSAT appeared to be a better differentiation between each proficiency cluster. High achievers in the English GSAT appeared to perform better on the GEPT listening and reading tests than did low achievers. Yet, apart from the GEPT reading test, the English GSAT yielded weak predictive power of student course attainment, which seemingly varied across classes, hinging on course designs and grading policies. Implications drawn from the results of the investigation were further discussed.

## Introduction

Between-class ability grouping practice has been widely implemented in English as a foreign language (EFL) tertiary education to cater to the needs of learners with varying English abilities. Recent years have witnessed extensive research on students’ or teachers’ attitudes toward the homogeneous placement (e.g., Chen, 2016; Joyce & McMillan, 2010; Kim, 2012; McMillan & Joyce, 2011; Sheu & Wang, 2006) or on the effects of ability grouping teaching on English learning (e.g., Liu, 2009; Sheppard et al., 2018). Yet, the question as to whether learners are accurately placed into appropriate levels of English classes remains under-researched, particularly in EFL settings.

Accurate placement is expected to lead to optimal teaching and learning as well as increase the likelihood of academic success, while misplacement may undermine the effectiveness of teaching and learning and put excess financial stress on educational institutions and students (Fox, 2009; Hille & Cho, 2020). Nevertheless, how to accurately place students according to their English proficiency has been a great challenge faced by educational practitioners (Su, 2010), for reliable and valid criteria for classification are difficult to attain (McMillan & Joyce, 2011), and students’ intentions of taking in-house placement tests are hard to control (Su, 2010).

In EFL ability grouping programs, learners are usually streamed into classes mainly based on their performance on English entrance exams, in-house English placement tests, or commercially produced standardized proficiency tests. With concerns about intensive resources required for placement test development and the high cost of standardized testing, many EFL programs tend to use students’ scores on admission tests for placement. English is a required subject for college entrance exams in many non-English speaking countries, notably in Asia, and has thus commonly been used as a convenient, economical, and credible placement tool.

Further, the spread of the COVID-19 pandemic has constrained higher education institutions from using standardized test scores to identify students with language support needs or delivering face-to-face placement tests (Ockey, 2021). Using college admission test scores for EFL placement purposes has consequently become a quick-fix for the critical issue confronted by many universities. A series concern, however, arises as to whether the accuracy and validity of placement decisions made using the admission test scores will be compromised.

In the context of placement decisions mainly based on test scores, the alignment between test task characteristics and the content of instruction is a determining factor in the effectiveness of placement decisions (Hille & Cho, 2020). A mismatch between what is assessed and what is taught may make it difficult to accurately inform placement decisions (Kokhan, 2013) or significantly predict student subsequent performance in the classes (Hille & Cho, 2020; Lee & Greene, 2007).

Addressing the validity concern is particularly critical for institutions which have long used national high-stakes entrance exams for placement purposes. These admission tests are not designed for placement use by local language programs and therefore fail to include test tasks and content resembling the ones that students may encounter in a real language course, i.e., the target language use (TLU) domain (Bachman & Palmer, 1996), resulting in the growing concern over the validity of the placement decisions made based on the test scores.

In such a case of tests whose scores are utilized not only to make inferences about students’ current language proficiency but also to predict their suitability for a future course or program, Field (2013) underlines the significance of cognitive validity, referred to as “the extent to which a test requires a candidate to engage in cognitive processes that resemble or parallel those that would be employed in non-test circumstances” (p. 78).

Motivated by Field’s (2013) notion of cognitive validity, this investigation attempted to delve into the accuracy and validity of placement decisions made based on college entrance exam scores in the context of the English General Scholastic Ability Test (GSAT), a nationwide high-stakes college admission test in Taiwan commonly used for EFL placement. The study has both theoretical and practical ramifications for L2 testers, educational institutions, teachers, and students, for empirical evidence will be provided to inform the stakeholders about adjustments to teaching and the policy and practice of ability group placement as well as to contribute to the existing testing research concerning the effectiveness of placement tests.

## Literature review

### Concerns over the effectiveness of placement tests

The central purpose of placement tests is to assess learners’ language proficiency so as to place homogeneous language-ability learners in appropriate language courses or classes (Hughes, 2003; Li, 2015; Weaver, 2016). Groupings can be made based on a student’s relative standing along the scoring continuum, referred to as a norm-referenced test (NRT), or depending on a student’s mastery or nonmastery of certain learning objectives, called a criterion-referenced test (CRT) (Green, 2012; Long et al., 2018).

Placement tests can also be categorized into the types of language course-based and proficiency-oriented tests (Wall et al., 1994). The first type “has a pre achievement orientation such as the English Placement Test at the University of Illinois at Urbana Champaign that reverberates the academic demands of the courses offered at the university” (Weaver, 2016, p. 6), whereas the second type has a general proficiency orientation, such as the General English Proficiency Test (GEPT), TOEFL, TOEIC, and IELTS, with no strong association with the content of language course.

Since the primary goal of a placement test is to correctly place a student into an appropriate class (Brown, 2004), it is of particular importance to examine its effectiveness in terms of the accuracy and validity of placement decisions made based on the test scores. Inaccurate placement tends to exert negative influence on teaching and learning, namely, harmful *washback* (also known as *backwash*), which is an aspect of impact of test use (Bachman & Palmer, 1996) and is connected with construct validity (Messick, 1996).

Differences in the test content and the objectives and academic demands of the course have been identified as a crucial factor in misplacement (McMillan & Joyce, 2011). One of the critical criteria for a placement test is, thus, to include test tasks which “enable valid inferences regarding mastery of knowledge, abilities or skills taught on the course” (Green & Weir, 2004, p. 469) so as to counter the two threats to construct validity known as “construct under-representation” and “construct-irrelevant variance*”* (Messick, 1996, p. 244). In this regard, to what extent the test task characteristics, including text and task features, align with the content of instruction emerges as an important indicator of the effectiveness of placement decisions (Hille & Cho, 2020).

To ensure that placement tests accord with the instructional content and objectives of language programs, some schools attempt to develop placement tests to correspond to curricular. However, some researchers (e.g., Kung & Wu, 2010) cast doubt on the reliability of these internally developed placement tests and raised concerns about the score-based inferences being made based on the test scores, which in turn imperil the accuracy of placement decisions. In Su’s (2010) survey with 452 college students, only 35.5% of the responses to using in-house placement tests to group students were positive.

Another challenge lies in students’ attitudes towards taking post-entry placement tests. Students’ intention to fail placement tests to obtain good grades or an easy pass invokes concerns over test fairness (Su, 2016). Furthermore, developing a high-quality placement test is rather time-consuming and resource-intensive. Without sufficient support and relentless research effort, it is not easy for a local language program to develop a credible placement test with high reliability and validity (Kung & Wu, 2010).

Given the insurmountable issues involved in self-designed placement tests and a plethora of time and human resources required for test development, there has unsurprisingly been an increased use of commercial standardized proficiency tests for placement, grounded in a belief that most commercially available standardized tests have been carefully developed and thus help to make correct placement decisions. A recent survey study (Ling et al., 2014) revealed that using commercially available tests for placement purposes have gained popularity.

Evidence was presented in the study of Wang et al. (2008) to favor using TOEFL iBT for placing ESL students. Papageorgiou and Cho (2014) also found a strong relation of secondary school students’ TOEFL Junior Standard scores to teacher judgments of placement levels and a moderate prediction accuracy of placement based on the test scores, ranging from 72% (for school B) to 79% (for school A).

Yet, not all proficiency tests can provide specific diagnostic information (Green & Weir, 2004). Standardized proficiency tests are usually designed to assess skills, knowledge, and abilities within a certain target language domain (Fulcher & Davidson, 2009), which may be different from students’ learning experience and environment (Weaver, 2016) or may fail to address specific needs of a particular language program (Kokhan, 2012). Such a mismatch may pose a threat to the validity of the interpretation of test scores and placement decisions afterwards.

Kokhan (2013) investigated the appropriateness of using pre-arrival standardized test scores for ESL placement and argued against this method as there was a high probability that students might be misplaced. In a similar vein, Fox (2009) found large variability among students placed in the same level of the English for Academic purposes (EAP) program using commercially available proficiency test scores and raised concern over misplacement problems, which led to a negative impact on teaching and prompted teachers to rely on additional information about their students so as to meet their needs.

Conflicting results in previous studies have attracted increasing research attention. Hille and Cho (2020) recently examined placement accuracy by comparing ESL students’ test scores on two commercially available tests and one locally developed writing test with their actual placement levels determined by their teachers. The results revealed that each test by itself performed similarly in predicting student placement and the combined use of all three tests produced the highest accuracy, yet neither individual tests nor any combinations of these tests could significantly predict students’ course grades and advancement to the next level.

Hille and Cho’s (2020) study contributes to the understanding of the predictive value of commercial standardized tests and in-house placement tests in informing placement decisions and student subsequent performance in class. However, as most previous studies, the ESL context of their study where taking a standardized English proficiency test is usually a pre-entry requirement for matriculated international students may make it difficult to generalize the findings to the EFL context where students are generally required to take nationwide entrance exams developed based on the national curriculum standard.

In many non-English speaking Asian countries, English is a compulsory subject included in college entrance exams. Some schools, hence, tend to rely on students’ scores on the English admission test for placement into levels of English courses for economical and convenient reasons, a ubiquitous phenomenon in universities and colleges in Taiwan.

In Taiwanese higher education, ability grouping for English instructions has been encouraged to implement by the Ministry of Education since 2001 (Chien et al., 2002). The means of and criteria for placement yet vary across tertiary institutions (Su, 2010). Students may be placed into different levels of English class based on their performance on the English college entrance exams (Tsai, 2008), English proficiency tests (Liu, 2009), or language course-based placement tests (Tsai, 2008). Some schools may regroup students pursuant to their achievements during semesters (Yu, 1994). These placement practices are implemented and favored for different reasons. Given the popularity of using college entrance examinations for placement in EFL settings, the following discussion will center on relevant issues.

### Previous studies on the use of English college entrance examinations for EFL placement

In contrast to ESL settings where most international students are required to take standardized proficiency tests before entry to prove their English language ability, EFL learners in many non-English speaking countries encounter the challenge of taking a nationwide English admission test as a prerequisite for studying in college. The credibility and accessibility of college entrance exams, as well as the gaining popularity of ability grouping instructions, have made these admission tests become a prevalent alternative tool for placement in some Asian countries such as Japan (LeTendre et al., 2003) and Taiwan (Yang & Lee, 2014).

The practice of English ability grouping has been commonly implemented in many universities and colleges in Taiwan, with the use of students’ scores of college entrance exams for making placement decisions (Tsao, 2003). Yet, questions remain as to how well these college admission tests can function as placement tests. One particular area of concern is the accuracy of the score-based classification decisions and the predictive validity of these entrance exams in placement testing contexts. However, these issues have, regrettably, been insufficiently researched.

Feng and Chang (2010) compared 1511 Taiwanese non-English major freshmen’s test scores on the TVE (Technological & Vocational Education) Joint College Entrance Examination and on the Global English Test (GET) and found that the TVE English scores were reliable and useful to be used as a basis for grouping students and claimed that additional placement tests would not be necessary. Nevertheless, the feasibility of college entrance exams as placement tests was still not fully discussed.

Given the concern over the appropriateness of the English GSAT which does not include a listening component for placement into both English reading and listening courses, Yu (2009) compared 2347 college students’ English GSAT scores with their scores on self-developed English reading and listening tests (following the test format and difficulty level of the intermediate-level GEPT reading and listening tests). The English GSAT appeared to be highly correlated with the internally developed reading and listening tests, indicating that the English GSAT had a high level of criterion-related validity and worked well to stream students into different reading and listening classes. However, the content alignment between these tests and courses and the use of cut scores (Hille & Cho, 2020) were not thoroughly investigated.

Another related question emerges as to whether the English GSAT can still function well in differentiating between students for the second semester. Yu and her colleagues (Yu, 2009) continued, in the same study, to administer another form of reading and listening proficiency test to 2118 students who had completed their first-semester English course. The results revealed that the English GSAT had no significant correlation with the second proficiency test and neither did it have strong predictive power of the proficiency test scores, suggesting that the discrepancy between students’ proficiency had increased and thus warranted another administration of proficiency test for placement. Yet, plausible factors contributing to the non-significant correlation between these tests were not explored.

In Yang and Lee’s (2014) recent study, 805 Taiwanese college freshmen who took the required year-long English reading and writing course were placed into three levels based on their college entrance exam scores. They were given the Oxford Online Placement Test in the beginning of the first semester to assess their reading ability. Their test performance consistently varied across the three levels and moderately correlated with their college entrance exam scores. Yang and Lee contended that it seemed feasible to use college entrance exam scores for the reading and writing course placement. Slightly different from Yu (2009) though, they considered content alignment and treated the use of English GSAT for placement into English listening and speaking courses with caution.

Recently, Su (2016) also found a similar qualm expressed by EFL teacher participants in her survey study and argued that “students who score high on reading and writing tests may not necessarily perform well in speaking and listening skills” (p. 66). So a majority of respondents in Tsai’s (2008) survey study favored using both college entrance exam scores and internally developed placement test scores to place them so that their current language proficiency level could be more accurately assessed, resonating with some researchers’ (Hille & Cho, 2020; Kokhan, 2012) arguments for multiple measures.

Given that English college entrance exams aim to select prospective students for universities, not to place students into EFL classes for a specific local program, specific skills and dimensions of language competence assessed in these admission tests may not correspond to the teaching objectives and language skills demanded for a particular English course. The feasibility of using a college admission test for EFL placement is thus called into doubt and yet is insufficiently researched.

A complete picture of placement accuracy in terms of the relations of college admission tests to commercial or/and in-house tests as well as to student academic success has not yet been obtained. In particular, not much research to date has examined the relationships with concerns over content alignment and cut-off scores across levels of English classes or compared the relative predictive power of tests for student subsequent performance in EFL classes.

### The current research

As demonstrated in the preceding review, the appropriate use of tests for making fair and correct placement decisions is one of the decisive factors contributing to the success of ability grouping instruction. Notwithstanding the ubiquity of using high-stakes English college entrance exams as placement tools, research attempts into the feasibility and validity of these admission tests used for placement have thus far been remarkably scarce, particularly in EFL settings.

The purpose of the study, therefore, is two-fold: (a) to investigate whether a college admission test can serve as a fair and valid placement tool and its relations with other English proficiency tests across level classes and (b) to examine its predictive power, in comparison with other proficiency tests, on students’ performance in English classes. The specific research questions raised for this study are as follows:

1. To what degree can the English GSAT correctly place students into appropriate EFL classes? Specifically, what is the placement agreement between the English GSAT and GEPT?
2. How are the scores on the English GSAT and GEPT inter-correlated?
3. To what extent can the English GSAT, in comparison with the GEPT, predict students’ subsequent performance in EFL classes?

## Method

### The setting: Freshman English for non-majors program at the target school

The Freshman English for non-majors course (also referred to hereafter as “FE”) is a two-semester-long compulsory course (with three credits for each semester) for college freshmen within the institutional context of the present study. FE is offered to approximately 2500 new students annually at two levels: regular class and developmental class, both of which are two-semester-long and offered at the same time. Students’ scores on the college entrance exams, i.e., the English GSAT or/and the DRET (Department Required English Test), are used for making placement decisions.

It is worth noting that the majority of the students at the target school in Taiwan appear to be high achievers with an average English GSAT score and an average DRET score both at the 88th percentile, i.e., better than 88% of the rest of all the examinees in the country and thus will mostly be placed into regular classes, while the bottom 20% of their cohorts whose scores are in the lowest 20% among freshmen at that school will be required to take developmental classes. A ballpark figure of the class number is about 50 to 55 regular classes and 8 to 10 developmental classes every year, each with 25 to 35 students.

Despite the fact that all the teachers in the FE program have worked together to stipulate what teaching objectives and learning goals to be achieved, there is actually no coherent curriculum for either regular or developmental classes where the teachers have independently designed their courses and chose teaching materials they believe would be appropriate for their students’ ability level. Consequently, what students learn and are required to do as well as how their course achievements are evaluated may vary across classes.

Since each class may have disparate teaching objectives and demands, such variation between class calls into question the correctness and validity of placement decisions being made based on the students’ English GSAT or/and DRET scores. Both the English GSAT and DRET are college entrance exams but administered on different dates, with the same purpose of screening high school graduates for college admission, albeit there is a much larger proportion of students taking the English GSAT as compared to the DRET. In this target school, almost every student (about 99%) would take the English GSAT, but only half of the students (about 49%) would take the DRET or both tests. In view of this particular condition, the English GSAT is mainly used for placement at the target school. The current study, therefore, will only probe into the feasibility of the English GSAT as a placement test.

### Participants

The present study involved 124 EFL learners with Chinese as their native language enrolled at a university in northern Taiwan. However, 24 students were dropped due to missing information or ineligibility for participation, leaving a sample of 100 college freshmen used in the current investigation. Since the participants were naturally assembled in FE classes, cluster sampling was used with a class as the sampling unit (Wiersma, 2000).

The data were collected from four FE classes: two regular classes (including 55 students taught by two different teachers) and two developmental classes (including 45 students taught by the same teacher). Table 1 enumerates the number of students for each class and students’ colleges.

**Table 1:** Number of students for each class

Instructor	Class level	College	Student number
Teacher C	Regular	Science	29
Teacher M	Regular	Engineering, computer science, law	26
Teacher F	Developmental	Liberal arts	26
Teacher F	Developmental	Management, life science	19

### Instruments

A number of instruments were used for the current study, including the English GSAT and the GEPT High-Intermediate Level Listening and Reading tests. Information about the course designs of the four FE classes and the students’ final FE course grades were collected as well. The instruments and documentation used for this study are explicated as follows.

#### The English GSAT

The General Scholastic Ability Test (GSAT) is a high-stakes college entrance examination developed by the College Entrance Examination Center (CEEC) with the aim of assessing students’ basic knowledge and skills of required high school subjects and their readiness for college study (for more information about the GSAT, visit the website: https://www.ceec.edu.tw/).

The test is annually administered in January and is primarily used in the initial stage of screening. It is a required admission test for most high school graduates. Those who meet the required standards can go through the Personal Application process or can be recommended by their high schools to the universities of interest and proceed to the second stage, which usually involves interviews and document reviews.

A constant internal (for test reports, visit the CEEC website: https://www.ceec.edu.tw/) and external (e.g., Liao, 2015; Lin et al., 2018) research effort has been put into the investigation of the reliability and validity of the GSAT to ensure the usefulness of the test as a gatekeeper who determines potential candidates for admission.

The GSAT comprises five subject subtests: Chinese, English, Mathematics, Science, and Social Studies. The scaled score for each subject ranges from 0 to 15, with a total perfect score of 75. The English GSAT consists of vocabulary, cloze, and reading comprehension multiple-choice questions, a translation task, and essay writing (see Table 2). It serves as the major placement tool for the FE program at the target school and thus would be the primary focus of this investigation. The study participants’ English GSAT scores would be collected for subsequent analyses.

**Table 2:** The tests used for the current study

	English GSAT	GEPT listening	GEPT reading
Language skill measured	Lexico-grammar and reading (72%) Writing (28%)	Listening	Lexico-grammar and reading
Task type	Vocabulary Cloze Gap-filling reading Reading comprehension Translation Essay writing	Answering questions Conversations Short talks	Sentence completion Cloze Reading comprehension

#### The GEPT High-Intermediate Level Listening and Reading tests

The General English Proficiency Test (GEPT) is developed by the Language Training and Testing Center (LTTC) to assess EFL learners’ English proficiency for school admission purposes or screening job applicants. The test is a five-level criterion-referenced testing system: Elementary, Intermediate, High-Intermediate, Advanced, and Superior (Wu, 2012). Currently, the GEPT is the most widely taken English language test in Taiwan (Wu & Lee, 2017). Compelling evidence of reliability (e.g., Liao, 2016) and validity (e.g., Chan et al., 2014; Weir et al., 2013) has been accumulated over the past few years to support the test as a good indicator of learners’ English ability.

In light of the ubiquity of the GEPT as a placement test and the graduation benchmark (Wu & Lee, 2017), the current study adopted this test to measure the participants’ English ability in an attempt to compare the placement decisions with those made based on the English GSAT scores.

The GEPT High-Intermediate Level was used for this study. The test aims to evaluate learners’ command of English in dealing with a wider range of topics. It comprises four subtests: listening, reading, writing, and speaking (for more information about the GEPT, visit the website: https://www.lttc.ntu.edu.tw). Test-takers are required to pass the listening and reading subtests in the first stage prior to proceeding into the second stage which involves writing and speaking subtests. According to the LTTC (n.d.-a), those who pass the speaking and writing subtests tend to have a higher level of English proficiency than those who only pass the listening and reading tests.

The current study focuses only on the listening and reading subtests which correspond roughly to the CEFR (the abbreviation for “Common European Framework of Reference for Language: Learning, Teaching, Assessment”) B2 level, the graduation benchmark set by the target school of the study. As shown in Table 2, the listening subtest contains three tasks with 45 multiple-choice items: (1) answering questions (15 items), (2) conversations (15 items), and (3) short talks (15 items). The test time is 35 minutes. The reading subtest also includes three tasks with 45 multiple-choice items: (1) sentence completion (10 items), (2) cloze (15 items), and (3) reading comprehension (20 items). The test time is 50 min.

The cut-off score for passing the listening or reading subtest alone is 80 out of 120 total possible points (i.e., 66.67% of the items on each subtest answered correctly). If a test-taker takes both the listening subtest with a total possible score of 120 and the reading subtest with a total possible score of 120, then the cut-off score will be 160 out of 240 in the condition that no single subtest score is lower than 72.

#### Grading policies of the four classes and students’ final FE course grades

In the FE program at the target school, English teachers independently design their courses, choose learning materials, and determine how to evaluate their students’ learning outcomes. In other words, each class has diversified course demands, requirements, and grading policies, which in turn provokes concerns over the appropriateness of the English GSAT for placement within this context.

To address this issue, this study worked with four classes (two regular classes and two developmental classes) offered by three different English teachers. The researcher contacted the teachers and inquired about their course information. The three English instructors’ course syllabi and their students’ final course grades were collected and analyzed for this study.

Table 3 presents the grading policies of each class. All the four classes aim to enhance students’ English ability by integrating the four language skills into a variety of learning activities but come with different course demands and grading policies. Both teacher C and teacher M taught regular classes. Teacher C placed great emphasis on speaking and listening and evaluated her students learning achievements mainly through oral presentations, blog writing, and listening activities, while teacher M foregrounded the development of speaking and writing skills by primarily involving students in making oral presentations and writing scripts and essays. In spite of the slight difference in teaching activities, both regular classes mainly focus on the development and assessment of oral presentation skills.

**Table 3:** Grading policies of each class

Class	Grading policy
Teacher C: regular class	Attendance and participation: 35% Learning project (blogging, presentation, project report): 30% Listening quizzes: 20% GEPT listening and reading tests: 15%
Teacher M: regular class	Oral presentations and presentation scripts: 50% Online practices (integrating listening, speaking, and writing): 20% Vocabulary quizzes: 15% Essay writing: 15%
Teacher F: developmental class	In-class vocabulary, reading, and listening tests: 64% Speaking assessments (oral presentation, article discussion): 11% Assignments (online practices, essay writing): 18% GEPT reading and listening tests: 2% Class participation: 5%

Dissimilar to the aforementioned two regular classes, students in the two developmental classes offered by teacher F were required to take several in-class vocabulary, reading, and listening tests which account for most of their final course grades, accentuating the significance of reading and listening skills for developmental classes. Although the developmental classes also required students to make oral presentations, the presentation performance did not account for much of the students’ course grades.

### Data collection and analysis

All the three English teachers involved in this study received their students’ English GSAT scores in the beginning of the semester and thus could better understand their students’ learning background to address their needs. One form of the GEPT High-Intermediate Listening and Reading tests were also administered to their students assembled in the four FE classes so as to obtain more information about the students’ current level of English proficiency. At the end of the semester, the researcher invited the teachers to take part in this study and made inquiries about how they designed their courses and evaluated students’ course performance. The teachers’ course syllabi and their students’ final course grades were then collected and analyzed by a series of statistical analyses.

Descriptive statistics were first computed to examine the distribution and variability of the student participants’ test scores. Next, independent *t*-tests were adopted to examine the discrepancy in test performance between the developmental classes and regular classes (i.e., the two-way placement). One-way ANOVA was then employed to appraise the three-level placement decisions (a new placement system attempted in the current study by resetting the cut-off English GSAT scores), followed by the implementation of correlational analyses and multiple regression analyses in an attempt to determine any possible relationships between the students’ test performance and their academic performance as well as which, if any, of the test indicators significantly predicted the students’ course achievements and their relative contribution. All these statistical analyses were performed in IBM SPSS Statistics 22.

## Results

### Descriptive statistics

Descriptive statistics were first computed for the developmental classes and regular classes. As illustrated in Table 4, the English GSAT mean score of the developmental class students was 11.64, significantly much lower than the one of the regular classes (*t* = − 9.69, df = 55.39, *p* < 0.01). The perfect scaled score of the English GSAT is 15. Original raw scores range from 0 to 100, with the interval of 6.32 for each scale, that is, the discrepancy of 2.69 in the mean score between the developmental and regular classes represents a remarkable difference of 17 in the original raw scores.

**Table 4:** Descriptive statistics of the English GSAT, GEPT, and final course grades

	English GSAT Mean (Std. D)	GEPT listening Mean (Std. D)	GEPT reading Mean (Std. D)	Final course grade Mean (Std. D)
Developmental class (*N* = 45)	11.64 (1.75)	69.76 (22.59)	72.67 (19.94)	82.04 (12.58)
Regular class (*N* = 55)	14.33 (0.70)	92.89 (14.11)	101.11 (12.09)	84.60 (5.98)

Even more substantial differences in the GEPT test performance were also found between the developmental and regular classes (see Table 4). The regular classes significantly outperformed their developmental counterparts both in the GEPT listening subtest with a score difference of 23.13 (*t* = − 5.98, df = 70.70, *p* < 0.01) and reading subtest with a score difference of 28.44 (*t* = − 8.39, df = 69.33, *p* < 0.01). Yet, there was no significant difference in the average FE course grades between the developmental and regular classes (*t* = − 1.253, df = 60.08, *p*>0.05).

It is worth noting that the developmental classes evidently yielded a larger standard deviation in the test scores and final course grades than the regular classes, suggesting that there is a tremendous amount of variation among developmental class students in terms of their English language ability and English class achievement, calling into question the accuracy of the two-level placement being made by the English GSAT.

*RQ1: To what degree can the English GSAT correctly place students into appropriate EFL classes? Specifically, what is the placement agreement between the GSAT and GEPT?*

Following the 2005–2008 Administration Guidelines by the Ministry of Education (MOE) of Taiwan (see more discussions in Chu & Yeh, 2017), the target school of the current study set an English benchmark for college undergraduates with a threshold of CEFR-B2. The criterial proficiency level for regular classes approximates to B2, while developmental classes target students at the level of B1 or below. Those who meet the English proficiency level of B2 or above on the CEFR scale in all four skills can apply for an exemption for the Freshman English course.

The GEPT is one of the most common English proficiency tests taken by the target school students to meet the benchmark requirement for graduation or exempt from the English course. It has also been commonly used as a placement test. As such, to investigate to what degree the English GSAT can correctly place students into appropriate EFL classes, one form of the high-intermediate GEPT listening and reading tests was administered to the student participants so as to examine the placement agreement between the English GSAT and GEPT.

The regular classes were expected to have a high likelihood of passing the high-intermediate GEPT listening and reading tests which correspond to the CEFR B2 level, while the developmental classes were presumed to be below B2 with a high probability of failing the GEPT. The passing standard for the high-intermediate GEPT listening and reading tests is the total test score equal to or above 160 out of 240, with each subtest score no lower than 72 (LTTC, n.d.-b).

As presented in Table 5, the passing rate of the high-intermediate GEPT listening and reading for the developmental classes was 35.6%, as opposed to 87.3% of the regular classes. In other words, using the results of the GEPT for placement, 35.6% of the developmental class students might be considered misplaced. By contrast, only 12.7% of the regular class students failed the GEPT and thus might be reckoned misplaced.

**Table 5:** Passing rate of the GEPT for the developmental and regular classes

	Developmental class (*N* = 45)	Regular class (*N* = 55)
English GSAT cut-off score	13 or below	13-15
Student number of passing the GEPT	16 (35.6%)	48 (87.3%)
Student number of failing the GEPT	29 (64.4%)	7 (12.7%)

The results of a high passing rate for regular classes were not surprising, while the passing rate for the developmental classes was not expected to be so high. A plausible reason is a high variability in the proficiency levels of the developmental class students. Put it another way, the current cut-off score using the English GSAT for making two-level placement was able to identify high achievers. Still, it did not function well to differentiate low achievers in English. Given that the average score of the students in the target school on the English GSAT was 14.17 (a percentile ranks (PR) of 88), the English GSAT score of 13 (a PR of 75) was set as the threshold for the two-level placement. Those who scored below 13 on the English GSAT were lumped into the same group, resulting in a wide gap in their proficiency.

Another potential contributing factor is the slightly varying cut-off score across colleges to control the class size. Although the target school set the cut-off English GSAT score at 13 for the developmental classes, a few students who scored 13 (seven students in this case) were still placed into regular classes to keep the minimum student number of 30, which might exacerbate the discrepancy in students’ English proficiency and in turn pose a threat to the correctness of placement.

To address this issue, this study tried to reset the cut-off English GSAT score and make a three-level placement for the students in an attempt to create a more precise, consistent, and accurate placement. In light of a mean score of 11.64 for the developmental class students, compared with 14.33 for those in regular class (see Table 4), students were re-classified into three levels of FE classes, using the cut score of 11 and below for the low-intermediate class, the scores of 12 and 13 for the intermediate class, and the cut-off point of 14 and above for the high-intermediate class.

Table 6 shows that none of the low-intermediate students passed the high-intermediate GEPT listening and reading tests (passing rate: 0%). In comparison, a large proportion of the high-intermediate students passed the tests (passing rate: 91.7%). By contrast, the intermediate students only had a fifty-fifty chance to pass the test. The three-level placement using the English GSAT scores appears to highly concur with the decisions being made by the high-intermediate GEPT listening and reading tests and aligns with the proficiency level targeted by the GEPT tests, suggesting that the three-level placement results in an increase in the correctness of placement and works better than the current practice of two-level classification.

**Table 6:** Passing rate of the GEPT for the three-level placement

	Low-intermediate class (*N* = 12)	Intermediate class (*N* = 40)	High-intermediate class (*N* = 48)
English GSAT cut-off score	11 or below	12–13	14–15
Student number of passing the GEPT	0 (0%)	20 (50%)	44 (91.7%)
Student number of failing the GEPT	12 (100%)	20 (50%)	4 (8.3%)
GEPT listening mean score (Std.D)	52.67 (18.65)	77.40 (20.50)	94.17 (13.03)
GEPT reading mean score (Std.D)	57.17 (15.84)	80.70 (18.60)	102.44 (10.94)
Final course grades	73.25 (14.85)	85.00 (9.23)	84.71 (6.33)

**Fig. 1:**
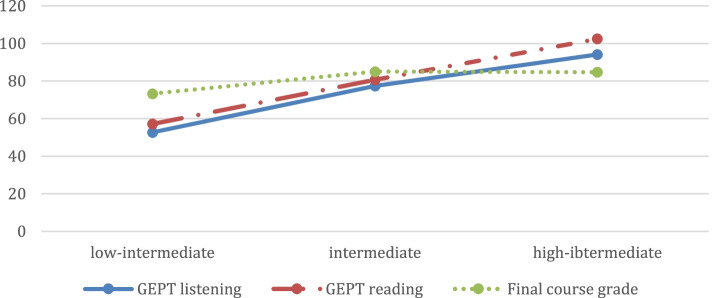
The GEPT listening and reading mean scores and the FE course grades for each level

As illustrated in Table 6 and Fig. 1, the high-intermediate class consistently scored the highest in the GEPT listening and reading tests, followed by the intermediate and low-intermediate classes. Interestingly, even though the English GSAT aims to evaluate high school graduates’ reading and writing abilities, excluding a listening component, the results of one-way ANOVA showed that the three-level classes regrouped by their GSAT scores significantly differed not only in their GEPT reading test performance (*F* = 52.189, *p* < .001) but also in their GEPT listening scores (*F* = 31.404, *p* < .001).

Post hoc tests were then performed using the Scheffé method to obtain specific information on which group means are significantly different from each other. It was found that the high-intermediate class scored significantly higher than the intermediate and low-intermediate classes on the GEPT listening and reading tests and that in the same vein, the intermediate class performed significantly better than the low-intermediate class in both tests, implying that the three-level grouping using the English GSAT could indeed differentiate students in terms of their listening and reading proficiency levels.

Another intriguing question is to examine whether high achievers in the English GSAT would be different from low achievers in their English course achievements. As discussed earlier, both developmental and regular classes produced similar mean scores of students’ final course grades. However, the three-level classes identified by using the English GSAT scores were again found to be significantly different in student English course achievement (*F* = 9.023, *p* < .001). The Scheffé test yet showed that although the final course grades of low-intermediate students were significantly lower than those of intermediate students and those of high-intermediate students, the intermediate and high-intermediate groups were not different from each other in their course performance, which indicates that other factors might exert influence on their learning and thus close up the gap between them.

*RQ2: How are the scores on the English GSAT and GEPT inter-correlated?*

Given the concern over the English GSAT’s criterion-related validity, a series of Spearman correlational analyses were subsequently conducted to inspect the relations of the English GSAT scores with the GEPT listening and reading test scores as the evidence of the concurrent validity. As can be seen in Table 7, the English GSAT was moderately^1^ correlated with the GEPT listening test (γ_s_ = .593) and strongly correlated with the reading test (γ_s_ = .710), each at the significant level of .01, signifying a moderately high level of agreement between the English GSAT and GEPT scores. In other words, how the students performed on the English GSAT was moderately highly related to their performance on the GEPT.

**Table 7:** Correlations among the English GSAT scores, the GEPT listening scores, the GEPT reading scores, and the final course grades

	All four classes (*N* = 100)		
	English GSAT	GEPT listening	GEPT reading
English GSAT	1	.593^**^	.710^**^
GEPT listening		1	.760^**^
GEPT reading			1
	Developmental class (*N* = 45)		
	English GSAT	GEPT listening	GEPT reading
English GSAT	1	.321^*^	.486^**^
GEPT listening		1	.659^**^
GEPT reading			1
	Regular class (*N* = 55)		
	English GSAT	GEPT listening	GEPT reading
English GSAT	1	.413^**^	.333^*^
GEPT listening		1	.602^**^
GEPT reading			1

*p < .05, **p < .01

Table 7 also illustrates that the English GSAT scores were significantly correlated with the GEPT reading scores (γ_s_ = .486) and with the GEPT listening scores (γ_s_ = .321) for the developmental class students. Similarly, significant yet not large correlation coefficients of .333 and .413 were produced for the regular class students’ English GSAT scores with the GEPT reading and listening scores, respectively, indicating some but not strong evidence of concurrent validity was found to support the English GSAT.

Another intriguing question may arise as to whether there is a varying agreement level across the three proficiency levels, as identified earlier. As shown in Table 8, the English GSAT scores were significantly and highly correlated with the GEPT reading scores for the low-intermediate learners (γ_s_ = .702) and with the GEPT listening scores for the high-intermediate learners (γ_s_ = .432). At the same time, no relationship was found between the English GSAT and the GEPT for the intermediate learners. In other words, for the intermediate learners, as their English GSAT scores increased, their GEPT scores did not tend to increase, denoting that the English GSAT may not well account for the variance in the intermediate leaners’ GEPT test performance. The result reverberates to the aforementioned concern over the accuracy of placement decisions using the English GSAT for the middle echelons of students.

*RQ3: To what extent can the English GSAT, in comparison with the GEPT, predict students’ subsequent performance in EFL classes?*

**Table 8:** Correlations among the English GSAT scores, the GEPT listening scores, the GEPT reading scores, and the final course grades across three levels

	Low-intermediate (*N* = 12)		
	English GSAT	GEPT listening	GEPT reading
English GSAT	1	.223	.702^*^
GEPT listening		1	.379
GEPT reading			1
	Intermediate (*N* = 40)		
	English GSAT	GEPT listening	GEPT reading
English GSAT	1	− .042	.171
GEPT listening		1	.636^**^
GEPT reading			1
	High-intermediate class (*N* = 48)		
	English GSAT	GEPT listening	GEPT reading
English GSAT	1	.432^**^	.277
GEPT listening		1	.537^**^
GEPT reading			1

To explore the predictive power of the English GSAT and GEPT on students’ subsequent performance in different ability-level English classes, a set of regression analyses using the standard multiple regression techniques were then separately employed in developmental and regular classes.

For multiple regression analysis, the English GSAT scores^2^, the GEPT listening scores, and the GEPT reading scores were treated as the explanatory variables, while the FE final course grades were designated as the dependent variable. Then, scatter plots of residuals and histograms of residuals were first inspected to warrant linearity, normality, and equal variance. The values of tolerance and variance inflation factor were also examined to ensure no violation of collinearity.

As shown in Table 9, the regression results for the developmental classes revealed that the model with the English GSAT, GEPT listening, and GEPT reading as predictors produced a coefficient of determination *R*^2^ of .348; that is, approximately 34.8% of variance in the FE course achievements was accounted for by the English GSAT, GEPT listening and reading scores (*F*(3, 41) = 7.299, *p* < .001). Of the three predictors, the GEPT reading (*β* = .380) had the most potent predictive power on the FE course performance, followed by the English GSAT (*β* = .246) and the GEPT listening (*β* = .058). Despite the fact that the direction of influence for all variables was positive, which means the higher scores on these tests the test-takers obtained, the higher grades for the English courses they received, only the GEPT reading scores appear to be a strong predictor at a significant level (*t* = 2.122, *p* < .05).

**Table 9:** Regressions for the predictors across classes

Variable	Developmental class	Regular class
Intercept	41.795*** (3.910)	46.792** (2.862)
GSAT	.246 (1.635)	.252 (1.732)
GEPT listen	.058 (.339)	.127 (.747)
GEPT reading	.380* (2.122)	.035 (.209)
*R*^*2*^	.348	.118
Adj *R*^*2*^	.300	.067
F ratio	7.299***	2.284
df	3, 41	3, 51

***P value < 0.001, **P value < 0.01, and *P value < 0.05

Values in parentheses are *t* values.

By contrast, only 11.8% of the FE course grade variance for the regular classes was explained by the three predictors, as can be seen in Table 9 (*F*(3, 51) = 2.284, *p*>.05). None of the three predictors exert significant influence on students’ final course grade, indicating extremely weak predictive power of the English GSAT and GEPT on the regular class student achievement, which might, in part, due to the regular class intense demand for oral presentation skills, an element excluded from the two tests.

## Discussion

The current study aimed to investigate the feasibility of the English GSAT as a placement test for an EFL program at a university in Taiwan. Developmental and regular classes were identified using the English GSAT scores. Substantial differences in the English GSAT and GEPT were found between the developmental and regular classes. The discrepancy between the two-level classes was notably more significant in reading than in listening, which reverberates with what was found in the English GSAT and might partly be attributed to the neglect of the listening skill and the overemphasis on reading instruction in high school English education.

By contrast, no significant difference was found in the average English course grades of developmental and regular class students. It suggests that the teachers opted for a criterion-referenced approach to evaluate their students’ learning outcomes without adjusting scores or delimiting top-ceiling scores according to streamed classes, which seems to be different from some schools where the grading system varies across levels (Su, 2010).

Note that in comparison with the regular classes, there appears to be a larger variation in developmental class students’ proficiency, which indicates a cause for concern over the placement accuracy, a predicament facing many schools with ability grouping programs (McMillan & Joyce, 2011). Contributing factors in placement results may include the use of cut scores (Hille & Cho, 2020) and non-score consideration such as class sizes (Plakans & Burke, 2013), as found in the study context.

This investigation thus examined the placement agreement between the English GSAT and GEPT and noticed that about one-third of developmental class students seemed to be misplaced, which might, in part, be due to the great variability in the students’ proficiency and the inappropriate use of cut-off score. In particular, those whose scores are close to the cut point between the levels would be more likely to be misplaced (McMillan & Joyce, 2011).

A three-level placement was then made in the study by resetting the cut-off scores and re-classifying the students into three proficiency-level classes. It was found that there was a high level of agreement in the three-level placement using the English GSAT and the decisions being made based on the GEPT listening and reading tests scores. In other words, the three-level placement was proved to be more effective and accurate in streaming students than the current two-level practice, mirroring some previous studies where most students approve of the three-level grouping practice (Su, 2010) and the English GSAT works well in placing students into three English reading proficiency levels (Yang & Lee, 2014; Yu, 2009). As pinpointed by McMillan and Joyce (2011), “reliability, validity, and program management decisions conspire to reduce placement accuracy. One approach to improving the allocation of students to classes would be to stream students more tightly” (p. 77).

Besides, a significant correlation was also found between the English GSAT scores and the GEPT listening and reading scores, indicating a concurrent validity of the English GSAT with the GEPT. The results were not surprising, for both tests are intended to measure students’ general English proficiency and contain components of lexico-grammatical knowledge and reading comprehension. Despite that no listening section was included in the English GSAT, a moderate correlation was still found between the two tests, which seems probable, for both involve comprehension skills. The results were in accordance with Yu’s (2009) study showing the English GSAT was highly correlated with the reading and listening proficiency test mimicking the GEPT.

Nevertheless, it should be noted that there is an eight-month time gap between the administrations of the GSAT (in January) and GEPT (in September), which might result in a lower correlation between these tests. As found in Kokhan’s study (Kokhan, 2012), the correlation between the TOEFL iBT and the EPT appeared to be stronger when the time gap was short, suggesting a post-entry test would help to better appraise students’ current language ability level and learning needs.

It is worth noticing that the English GSAT is fundamentally dissimilar to the GEPT in test purpose, test content, and task type. Even though the three-level placement made by the English GSAT concurs with the classification using the GEPT and is thus proved to function well, it is challenging to avoid misplacement altogether. As such, a combined use of multiple measures such as locally developed placement tests and teacher judgments may increase the placement accuracy (Hille & Cho, 2020). Also, adaptive course designs and appropriate material selection is of particular importance (Yang & Lee, 2014).

Furthermore, concurring with previous research (Hille & Cho, 2020; Lee & Greene, 2007) where placement tests did not significantly predict students’ academic success, the findings of the study revealed that of the English GSAT and GEPT scores, only the GEPT reading scores could predict developmental class student course performance, which might partly be attributed to the course design of developmental classes with a particular focus on reading and the grading policy where reading tests account for a large portion of a students’ final grade. By contrast, the regular classes place more emphasis on oral presentations, academic listening, and class participation, which are, in fact, not included in the English GSAT or GEPT. This finding could be interpreted positively, for students, if accurately placed, were expected to have an equal chance at academic success (Hille & Cho, 2020). As contended by Sheppard et al. (2018), the curriculum for different ability groups is usually adjusted to suit the diverse needs of learners and thus differs across classes. Such curriculum differentiation between the ability groupings might lead to different results with regard to the predictive power of the admission and proficiency tests.

Since college entrance exams and proficiency tests aim to evaluate students’ level of language ability, “they may fail to fully meet all the specific language needs of a certain program or educational institution” (Kokhan, 2012, p. 292) and accurately assess or predict students’ actual language performance in class. Doubts have been raised as to whether these tests could assess the incremental advances in learning within a particular program (Fox, 2009). Moreover, other crucial factors such as learning motivation and effort and individual cognitive ability might also come into play in determining student achievement (Hille & Cho, 2020; Lee & Greene, 2007; Renandya, 2013). Many researchers (e.g., Fox, 2009; Liu, 2009; Yu, 2009; Yang & Lee, 2014), therefore, foregrounded individual differences and the necessity of periodically assessing students’ progress after ability placement and then regrouping them.

In a nutshell, the findings of the study resonate with the call from some researchers (e.g., Hille & Cho, 2020; Kokhan, 2012) to use other supplementary evidence of English proficiency in addition to admission test scores for placement and invoke the concern over the feasibility of the GSAT as a sole placement test for all students with varying English ability.

## Conclusions

English placement testing is a prevalent testing event for streaming students into appropriate classes and often exerts a tremendous impact on students’ learning. Yet, not much research has been conducted on English placement tests in the field of language testing (Li, 2015). The feasibility of using college entrance exams for placement in EFL programs is particularly under-researched. The current study attempted to fill the gap in the context of the English GSAT. The results of this investigation give rise to both theoretical and practical implications summarized as follows.

First, the common policy of using college entrance exams to place students into EFL classes must be critically appraised for validity concerns. As defined by Messick (1989), validity refers to “the degree to which empirical evidence and theoretical rationales support the adequacy and appropriateness of interpretations and actions based on test scores” (p. 6). Little evidence has thus far been collected to support the use of college admission tests for EFL placement. Drawing on Messick’s (1989, 1996) view of construct validity and Fields’ (Field, 2013) notion of cognitive validity, the current study corroborated the concern over the degree of correspondence between TLU domains and language behavior elicited from the tests which may jeopardize the validity of the score-based inferences and decisions and demonstrated the importance of gathering empirical evidence to support construct validity.

More recently, increasing attention has been paid to a contemporary argument-based approach which focuses on identifying types of claims or inferences with regard to test score interpretation and use (i.e., *interpretive argument*) and collecting evidence to support or challenge the claims and inferences (i.e., *validity argument*) (Bachman & Palmer, 2010; Chapelle et al., 2008; Kane, 2013). This investigation implied the urgency of making validation efforts to construct arguments relating to specific inferences and evaluate them with backing or rebuttal empirical evidence (Kane, 2013) for the use of college entrance exams for EFL placement.

From a practical perspective, as found in this study, the English GSAT mainly measures high school graduates’ lexico-grammatical knowledge, reading ability, and writing ability and fails to indicate their English listening and speaking ability, two essential skills at the tertiary level. Collecting more evidence of proficiency is critical to compensate for the insufficiency of admission tests (Kokhan, 2012). Other measures of language proficiency such as post-admission proficiency tests (Kokhan, 2012; Wall et al., 1994; Yang & Lee, 2014) and/or self-rated English proficiency (Li, 2015; Ross, 1998) can help to provide more information about students’ language ability and make more accurate placement decisions.

What is more, “the determination of the cut-off points for the grouping arrangement needs to be carefully considered” (Liu, 2009, p. 143). This study found that students whose scores were close to the cut point were more likely to be misplaced. An inaccurate set of the cut-off score may exacerbate the variability between students, which may, in turn, affect placement accuracy (Hille & Cho, 2020) and the effectiveness of ability grouping instruction (Sheppard et al., 2018). In fact, a more tight and precise placement can help to increase the homogeneity of students grouped into the same class and consequently lead to a more accurate placement (McMillan & Joyce, 2011). Coinciding with previous findings (Su, 2010), this study found that a three-level placement functioned better in differing students with varying language abilities than did the two-level classification.

Despite that this investigation bridged the gap in the accuracy and appropriateness of using a college entrance exam for EFL placement and provided useful information for test stakeholders, it is still subject to some limitations, given that the study was conducted in a unique EFL context with a small group of students. The English GSAT appeared to be effective for placement in the study context; however, considering the “highly context-dependent nature of the placement process” (Hille & Cho, 2020, p. 457), the results of the study cannot be directly generalized to other programs with different curricular designs and test use. Also, in this study, student course achievement was mainly measured using students’ final course grades, which might result in uncertainty of what particular language skills are demanded by the course and to what degree college entrance exams correspond to language skills required for an EFL class. Further analysis by decomposing student course achievement into separate components may help address this concern.

Furthermore, learner affective factors such as motivation, anxiety, and attitude might influence the learning outcome (Fox, 2009; Kelsen & Liang, 2012), yet not taken into account in this study, and are suggested for future research to compare their relative explanatory power with the admission tests.

Some recent research sought to probe into teachers’ (e.g., Nasem, 2018) or students’ views (e.g., Kusaba, 2017) to appraise the effectiveness and appropriateness of a placement test. Further research is also warranted to quantitatively and qualitatively investigate and compare teacher and student perspectives on the use of college entrance exams for placement and placement decisions being made (McMillan & Joyce, 2011).

Continuing research endeavors to probe into the accuracy and appropriateness of using a college entrance exam for post-entry EFL placement will advance the understanding of this critical issue and engage more public attention to the consequential validity of test use and test-based accountability.

## Data Availability

The datasets used and/or analyzed during the current study are available from the author on reasonable request.

## Footnotes

1 According to Schober, Boer, and Schwarte (2018), a correlation coefficient can be interpreted as follows: 0.00-0.10 negligible; 0.10-p.39 weak; 0.40-0.69 moderate; 0.70-0.89 strong; 0.90-1.00 very strong.

2 Following the views of many researchers (e.g., Sullivan & Artino, 2013; Zumbo & Zimmerman, 1993) who regard a variable with five or more categories as an ordinal approximation of a continuous variable and argue the appropriate use for parametric tests, the English GSAT scores with 15 levels would be treated as a continuous variable so as to better interpret the regression results.
